# Joint effects of advancing age and number of potentially inappropriate medication classes on risk of falls in Medicare enrollees

**DOI:** 10.1186/s12877-019-1202-3

**Published:** 2019-07-19

**Authors:** Nicole K. Early, Kathleen A. Fairman, Jacqueline M. Hagarty, David A. Sclar

**Affiliations:** 1 Department of Pharmacy Practice, College of Pharmacy, Midwestern University, 19555 N 59th Avenue, Glendale, AZ 85308 USA; 2 Present address: Banner - University Medicine Diabetes and Endocrinology Institute, Phoenix, AZ 85006 USA

**Keywords:** Fall risk, Older adult, Potentially inappropriate medication, Elderly

## Abstract

**Background:**

Injurious falls among older adults are both common and costly. The prevalence of falls is known to increase with age and with use of fall-risk drugs/potentially inappropriate medications (FRD/PIM). Little is known about the joint effects of these two risk factors.

**Methods:**

Data for 2013–2015 were obtained from the Truven Health MarketScan® Medicare database comprising utilization and eligibility (enrollment) data for approximately 4 million enrollees annually. A case-control design was used to compare enrollees aged 65–99 years diagnosed with > 1 fall event (*n* = 110,625) with enrollees without falls (n = 1,567,412). An exploratory analysis of joint age-FRD/PIM effects on fall risks was based on number needed to harm (NNH) calculations for each FRD/PIM therapy class count (compared with 0 FRD/PIMs), stratified by age group. Logistic regression analyses adjusted for demographics, comorbidities, and fracture history, measured in the 1 year prior to the fall date (cases) or a randomly assigned date (controls).

**Results:**

For each FRD/PIM class count, NNH values decreased with older age (e.g., for 1 FRD/PIM class: from NNH = 333 for ages 65–74 years to NNH = 83 for ages 90–99 years; for 2 FRD/PIM classes: from NNH = 91 for ages 65–74 years to NNH = 38 for ages 90–99 years). NNH decreased to < 15 patients at > 6 classes for age 65–74 years, > 5 classes for age 75–84 years, and > 4 classes for age 85–99 years. Adjusted odds of falling were increased for age-FRD/PIM combinations with smaller NNH values: adjusted odds ratio (AOR) = 1.127 (95% confidence interval [CI] = 1.098–1.156) for NNH = 83–91; AOR = 1.427 (95% CI = 1.398–1.456) for NNH = 17–48; AOR = 1.983 (1.9034–2.032) for NNH < 15.

**Conclusion:**

FRD/PIM use and age appear to have joint effects on fall risk. Older adults at high risk, indicated by small NNH, may be appropriate for fall prevention initiatives, and clinicians may wish to consider decreasing the number of FRD/PIMs utilized by these patients.

**Electronic supplementary material:**

The online version of this article (10.1186/s12877-019-1202-3) contains supplementary material, which is available to authorized users.

## Background

Among older adults in the United States, fatal and nonfatal injuries from falls are both common and costly. The Centers for Disease Control and Prevention (CDC) estimates that more than 3.2 million falls occurred among U.S. adults aged 65 years or older in 2012, at a total direct medical cost of approximately $31 billion [1]. In 2014, 29% of older adults reported at least one fall in the previous year, and 38% of those indicated that the fall required medical treatment or prevented them from performing their usual activities for at least one day [2]. Fall risk increases with age and is generally greater among women than men; however, fall risk is particularly high for males aged 75 years or older [2, 3].

Use of certain fall-related drugs (FRDs), including benzodiazepines, antipsychotics, selective serotonin reuptake inhibitors (SSRIs), opioids, and some cardiovascular medications, is a known risk factor for the prevalence and severity of falls in older adults [4–7]. These medications are included in one or both of the most common potentially inappropriate medication (PIM) lists for older adults, both of which were updated in 2015: American Geriatrics Society Beers Criteria for Potentially Inappropriate Medication (PIM) Use in Older Adults (Beers List) and Screening Tool of Older Persons Potentially Inappropriate Prescriptions (STOPP) [8, 9]. Generally, the degree of fall risk increases in a dose-dependent manner with number of FRD/PIM therapy classes, although specific FRD/PIM definitions and cutoff values for risk classifications have varied considerably across studies [4–7, 10–12].

Building on this knowledge of fall risks associated both with aging and with FRD/PIM use, and on awareness that older adults commonly use multiple medications in compliance with evidence-based treatment guidelines, [13] a growing body of work has begun to use multivariate modeling to identify subgroups of FRD/PIM users at the highest risk of falling [14, 15]. The intent of this and similar modeling work is to target fall prevention efforts to those most in need of them [11, 12, 14, 15]. In this context, knowledge about the *joint* effects of age and number of FRD\PIM therapy classes on fall risk would be helpful. For example, this information could indicate whether use of > 2 FRD/PIM classes or > 4 FRD/PIM classes, typical cutoffs in many risk assessments, [12–14, 16] carries a greater risk for adults in some age groups than in others. Moreover, unlike some other known predictors of fall risk (e.g., indices of frailty or patient-reported measures), [12, 17, 18] these two risk factors are documented and easily retrievable in most automated health care payer databases.

However, most previous work has treated advancing age and FRD/PIM/FRD use only as *independent* risk factors, by measuring the effect of FRD/PIM use controlling for age as a confounder, without testing interaction terms for their potential *joint* effects. An exception is a study of the joint effects of number of medication classes and age group in a Taiwanese sample, which found that the degree of FRD-associated increase in fall risk was greater for those aged 75 to 84 years than for those aged 85 years or older [15]. However, that study used a broad list of medications, rather than those most strongly associated with risk of falling; and its outcome measure was limited to falls with fracture, not all falls requiring medical attention.

The present study addressed this gap in available in available information by developing and testing a method to quantify the joint (interactive) effects of FRD/PIM use and advancing age on fall risks, controlling for the independent effects of each factor.

## Methods

The study was a retrospective case-control analysis of de-identified data for Medicare enrollees aged 65–99 years. Case-control designs are commonly used to study medication-associated risk factors for adverse health events [19, 20]. The study was deemed exempt from Institutional Review Board (IRB) review by the Midwestern University IRB Committee.

### Data source

Data were obtained from the Truven Health MarketScan® Medicare database, which comprises health care service claims (i.e., billing) and eligibility (i.e., plan enrollment) data for approximately 4 million individuals enrolled each year with Medicare Supplemental insurance provided by employers. The database is fully Health Insurance Portability and Accountability Act (HIPAA)-compliant and includes both the Medicare-paid and employer-paid portions of services and payments. The health care claims included in the database represent all services and settings, including inpatient and outpatient hospital stays and visits; medications dispensed by community and mail order pharmacies; and ambulatory care (e.g., physician office visits, ambulatory surgical centers, laboratory, and radiology). Data are obtained by Truven Health, cleaned for quality and accuracy, and de-identified for research purposes. Data used in the study were for dates of service from January 1, 2013, through September 30, 2015.

### Sample inclusion and exclusion criteria

**Fig. 1:**
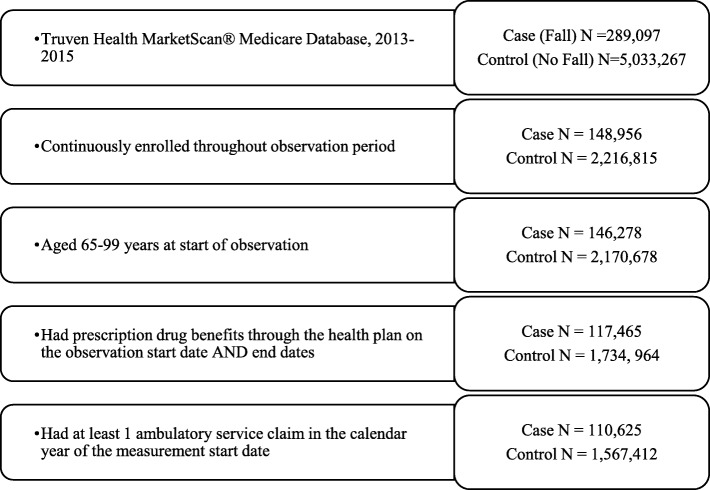
Sample selection flowchart

The sample comprised (1) cases with falls, defined as International Classification of Diseases, 9th Revision (ICD-9) codes of E880-E885 (accidental falls); E886-E888 (falls from collision with another person, other falls); and E987 (falling from a high place) and (2) control (i.e., no fall) enrollees (Fig. 1). Falls were identified for all medical settings of care. Diagnoses were measured in the first 4 diagnosis fields for all settings, plus the principal diagnosis field for all inpatient stays. The principal diagnosis field represents the primary reason for an inpatient admission, which typically is the discharge diagnosis.

For each calendar day and enrollee, records from all settings were merged, so that each daily record indicated whether that enrollee had a fall that was recorded in any setting on that date. Records were then aggregated to the individual enrollee level, and the first (earliest) fall date reported on a claim from any setting was identified for all cases with a fall. Each enrollee was then matched to his or her corresponding eligibility record, which indicated dates of Medicare enrollment. Any subject who had an eligibility record but no evidence of a fall was classified in the “no fall” control group.

All subjects (cases and controls) met the following criteria: (1) Continuously enrolled throughout an observation period that is described below; (2) aged 65 to 99 years at the start date of the observation period; (3) had prescription drug benefits through the employer; and (4) had at least 1 ambulatory service claim (i.e., physician’s office, urgent care, or clinic) during the observation period.

### Observation period

For all subjects, potential predictors of falls, including diagnoses and FRD/PIMs, were measured for the 365 days prior to an index date, allowing 7 measurement days after the index date to account for date of service discrepancies (e.g., laboratory test performed on one date but read and billed at a later date). For cases, the index date was the earliest fall date. For controls, the index date was assigned using random number generation. Specifically, the distribution (median and interquartile range) of number of days from enrollment start date to fall date was measured for fall cases. A random number range was established to mirror that distribution, and a random number of days within that range was generated for each control subject. The index date for each control subject was his/her enrollment start date plus the random number. After completion of this process, the mean observation start dates were November 4, 2013, and November 1, 2013, for cases and controls, respectively.

### Predictors

Potential predictors of falls were based on research literature documenting causes of falls in older adults [8, 9, 14, 21–29]. In addition to diagnoses (Additional file 1) and FRD/PIM therapy classes (Additional file 2) known to be potential causes of falls, [8, 9, 14, 21–29] potential predictors included fractures occurring from 365 days through 31 days prior to the index date. This pre-index time period was chosen to avoid measuring fractures occurring prior to the case fall date (i.e., predictor events) as fall/fracture outcome events. Fractures were measured as ICD-9 codes 800–804 (fracture of skull), 805–809 (fracture of spine and trunk), 810–819 (fracture of upper limb), and 820–829 (fracture of lower limb). To test the sensitivity of the findings to time period chosen for measurement of FRD/PIM therapy class use, a sensitivity analysis measured FRD/PIM therapy class counts in the 90-day, rather than 365-day, time period prior to the index date.

### Analytic procedures

Consistent with guidance for analysis of extremely large samples [30], greater emphasis was placed on clinical significance than on statistical significance in interpreting the findings, as a sample size exceeding 1.5 million subjects produces statistically significant results for all comparisons. In descriptive bivariate analyses, prevalence rates (i.e., percentages) for cases and controls, respectively, were calculated for demographic characteristics, diagnoses, and medication utilization during the observation period. In the descriptive analyses, unadjusted ORs (UORs) were calculated as the ratio of the odds (probability÷[1 minus probability]) for those with versus without each risk factor (e.g., odds of a fall for those with vs. those without atrial fibrillation). Age groups were categorized as follows, based on previous research on falls in older adults [4, 31]: 65–74 years, 75–84 years, 85–89 years, 90–94 years, and 95–99 years. Total counts of FRD/PIM therapy classes were summed across all drugs. For example, use of 2 opioid drugs, 2 antidepressants, and 1 benzodiazepine yielded a total FRD/PIM therapy class count of 3.

To assess the joint effects of FRD/PIM use and advancing age, number needed to harm (NNH), a standard measure of treatment-associated adverse event risk, [32, 33] was calculated for sample subgroups based on combinations of age and FRD/PIM therapy class count. NNH calculations were used because no quantitative information was available a priori to indicate specific cutpoints for combinations of age and FRD/PIM class count.

To calculate the NNH values, the percentage of subjects experiencing a fall was calculated for each combination of age group and counts of FRD/PIM therapy classes (e.g., 0 classes aged 65–74 years, 0 classes aged 75–84 years, etc.). Then, within each age group, for each FRD/PIM therapy class count of 1 or more, the absolute change in fall rate was calculated as the fall rate for that FRD/PIM therapy class count, minus the fall rate for 0 (zero) FRD/PIMs. NNH was calculated, using the standard formula, as the multiplicative inverse of the absolute change amounts, indicating the total number of subjects treated with each FRD/PIM therapy class count, instead of 0 (zero) FRD/PIMs, to produce 1 additional fall [34]. For example, for those aged 65–74 years, the percentages with a fall at 0 and 1 FRD/PIM therapy class were 2.2 and 2.5%, respectively. The corresponding NNH is 1÷(0.025–0.022) = 333, meaning that if 333 patients aged 65–74 years are treated with 1 FRD/PIM class instead of 0 FRD/PIM classes, 1 additional fall would be expected.

For the NNH calculations, some age-PIM class categories were combined to ensure that each NNH was based on at least 30 subjects. Specifically, the top 2 age groups (90–94 years, 95–99 years) were combined into a single group, representing those aged 90–99 years; and all FRD/PIM therapy class counts of > 10 were combined into a single category.

### Multivariate adjustment

To provide adjusted estimates, logistic regressions of the binomial fall measure (case vs. control) on predictors were performed in phases that reflected different approaches to measuring the effects of age and FRD/PIM use on fall risk. First, two models (Model 1 and Model 2) that treated age and FRD/PIM class use as independent predictors (i.e., as in previous work) were estimated to ensure similarity to previous results prior to testing the new, NNH-based approach. Then, to test the joint effects of age and FRD/PIM class count after controlling for the other known risk factors, the NNH was incorporated into a predictive statistical model (Model 3), a method that has previously been used to quantify medication-related risks [35, 36]. Results using the different approaches were compared using model chi-square, Nagelkerke R square, and the c-statistic (area under the Receiver Operating Characteristics curve) measure of predictive accuracy. Predictors for all equations included sex, diagnoses, and fractures treated in either inpatient or outpatient facilities.

In Model 1, which tested the effects of specific therapy class types, all therapy classes with UORs of > 1.5 were entered into the equation and removed using backwards stepwise regression (P in = 0.001, P out = 0.005). Because of the extremely large sample size, the UOR threshold of 1.5 was chosen as a benchmark for entry into the backwards stepwise equation based on previous studies of the same topic. These previous studies interpreted as clinically relevant adjusted ORs ranging from approximately 1.5 to 1.7 [19, 37, 38]. In Model 2, age and FRD/PIM use were also treated as independent predictors, but each subject’s counts of FRD/PIM therapy classes were summed and categorized as none, 1, 2, 3, 4, or 5 or more.

In Model 3, which used the new NNH-based approach, variables entered into the model were based on the NNH values obtained from the exploratory analysis: NNH of 200–333 (lowest risk, reference category); NNH of 83–91; NNH of 17–48; and NNH of < 15 (highest risk). Numeric gaps in the categories (e.g., 92–199) occurred because they were based on actual observed NNH values rather than a priori specifications.

Statistical significance of logistic regression coefficients was determined using 95% confidence intervals (CIs). All analyses were performed using SPSS (IBM SPSS, Armonk, NY) version 24.0.

## Results

Fall case subjects differed from controls demographically and on baseline (12-month pre-index observation period) medical diagnoses (Table 1). On average, case subjects were 6 years older (aged 80 years vs. aged 74 years, respectively), and a greater proportion of case subjects were female (63.4% vs. 54.1%). Diagnoses most predominantly associated with falls were, in descending order by UOR, dementia/mental impairment (UOR = 5.47), gait/balance disorders (UOR = 5.26), orthostatic hypotension (UOR = 4.20), Parkinson’s disease (UOR = 3.66), and dizziness/syncope (UOR = 3.36). Histories of inpatient stay and/or fracture multiplied the unadjusted odds of a fall by factors of 2.5–3.5.

**Table 1:** Baseline demographic and clinical characteristics, fall event cases and controls

	Case	Control	% with an Event	Unadjusted OR^a^
N (% of sample)	110,625	1,567,412 (93.4)	–	
Demographics				
Age mean (median)	80 (80)	74 (73)	–	
Age group (%)				
65–74 years	30.3	56.9	3.6	REF
75–84 years	38.4	31.6	7.9	2.30
85–89 years	19.0	8.0	14.4	4.50
90–94 years	9.9	2.9	19.3	6.40
95–99 years	2.3	0.5	23.5	8.23
Female (%)	63.4	54.1	7.6	1.50
Region (%)				
Northeast	21.1	24.3	5.8	REF
North Central	38.4	31.7	7.9	1.39
South	27.7	30.9	6.0	1.04
West	12.1	12.3	6.5	1.13
Unknown	0.7	0.9	5.1	0.87
Baseline diagnoses (%)^b^				
Atrial fibrillation	24.1	11.4	13.0	2.47
Cancer/malignancy	23.2	19.8	7.6	1.22
Dementia/mental impairment	20.1	4.4	24.5	5.47
Depression	18.2	7.6	14.4	2.68
Diabetes	32.0	26.8	7.8	1.30
Dizziness/syncope	27.3	10.0	16.1	3.36
Gait/balance disorders	22.8	5.3	23.1	5.26
Hepatic impairment	4.3	3.0	9.0	1.42
Hypertension	80.8	67.1	7.8	2.03
Neuropathy	17.8	10.7	10.5	1.81
Orthostatic hypotension	3.2	0.8	22.3	4.20
Parkinson’s disease	4.0	1.1	20.0	3.66
Renal impairment	17.9	9.1	12.2	2.18
Substance abuse	7.5	4.1	11.4	1.88
Vision disturbance/deficiency	46.9	43.9	7.0	1.12
Medical service use history (%)^c^				
Fracture history	9.1	3.8	14.4	2.50
Inpatient stays	22.7	10.5	13.2	2.52
For fracture	2.0	0.6	19.6	3.51
Ambulatory facility use^d^	57.4	41.5	8.9	1.90
For fracture	5.2	2.0	15.5	2.68

^a^Odds for case divided by odds for control, where odds = probability divided by 1 minus probability for group shown in row label. Reference group for female is male; reference group for regions is Northeast; reference group for all diagnoses is group without the diagnosis shown in the row label. ^b^Measured during the observation period. ^c^Measured from 12 months prior through 31 days before event or proxy date. ^d^Outpatient hospital, outpatient visit made in an inpatient hospital setting, emergency department, or ambulatory surgical center. *OR* odds ratio

Medications associated with fall risk included antipsychotics (UOR = 3.52 for first-generation, UOR = 3.25 for second-generation); antidepressants (UOR range from 1.55 for tricyclic antidepressants to 2.82 for monoamine oxidase inhibitors); other psychotropic medications (e.g., UOR = 1.63 for benzodiazepines); opioids (UOR = 2.01); and neuropathic medications (OR = 1.83; Table 2). Approximately one-quarter (24.7%) of cases, compared with 14.1% of controls, used > 1 opioid medications in the observation period. With the exception of loop diuretics (UOR = 2.39) and potassium-sparing diuretics (UOR = 1.69), antihypertensive medication use had no apparent association with fall risk.

**Table 2:** Use of potentially inappropriate medications in baseline period,^a ^fall event cases and controls

	Case %	Control %	% with an Event	Unadjusted OR^b^
Anticholinergic, all^c^	30.1	19.2	10.0	1.80
Anti-anxiety				
Benzodiazepine	21.5	14.2	9.6	1.63
Hypnotics	6.7	5.4	8.1	1.27
Antiarrhythmic (disopyramide)	0.0	0.0	9.7	1.52
Antidepressants				
MAOIs	0.1	0.0	16.6	2.82
SNRIs	7.0	3.7	11.9	1.98
SSRIs	23.9	12.4	12.0	2.21
TCAs	3.6	2.4	9.7	1.55
Other	10.3	5.2	12.2	2.07
Antiemetic	1.9	1.2	9.8	1.54
Antihistamine	6.6	4.7	9.1	1.44
Antihypertensive				
ACE inhibitors	26.2	23.4	7.3	1.15
Alpha 2 agonists	2.9	2.0	9.3	1.47
Alpha blockers	2.8	2.9	6.3	0.95
ARBs	15.1	13.4	7.4	1.15
Beta blockers	12.0	10.7	7.3	1.13
Calcium channel blockers	30.8	26.3	7.6	1.24
Central-acting	–	0.0	0.0	–
Loop diuretics	24.2	11.9	12.6	2.39
Potassium-sparing diuretics	4.4	2.7	10.5	1.69
Thiazide diuretics	11.6	11.2	6.8	1.03
Vasodilators	3.2	1.7	11.5	1.87
Antimuscinaric	9.2	4.7	12.1	2.05
Anti-Parkinsons	0.2	0.1	13.9	2.28
Antipsychotics				
First-generation	0.7	0.2	19.9	3.52
Second-generation	5.4	1.7	18.2	3.25
Anti-emetic/antipsychotic	1.2	0.7	10.5	1.66
Antispasmodic	4.9	3.9	8.1	1.27
Pain relievers				
Neuropathic	14.0	8.2	10.8	1.83
Opioid	24.7	14.1	11.0	2.01
Skeletal muscle relaxants	5.3	3.8	9.0	1.42

^a^At least one outpatient drug claim during the observation period. ^b^Odds for case divided by odds for control, where odds = probability divided by 1 minus probability for group shown in row label. Reference groups are those without use of the therapy class shown in the row label. ^c^See Additional file 2 for list of anticholinergic medications. *ACE* angiotensin converting enzyme, *ARB* angiotensin receptor blocker, *MAOI* monoamine oxidase inhibitor, *SNRI* selective norepinephrine reuptake inhibitor, *SSRI* selective serotonin reuptake inhibitor, *OR* odds ratio, *TCA* tricyclic antidepressant

NNH calculations indicated a joint (i.e., interactive) effect of age-FRD/PIM on fall risk (Table 3). Specifically, the degree of risk associated with increasing FRD/PIM use depended on age category, and the degree of risk associated with advancing age depended on FRD/PIM class count. For example, use of a single FRD/PIM therapy class was associated with relatively large NNH values for those aged 65–84 years (NNH = 333 for those aged 65–74; NNH = 200 for those aged 75–84), indicating relatively low fall risk. However, the risk of a fall associated with 1 FRD/PIM therapy class increased considerably among those aged > 85 years, as indicated by smaller NNH values (NNH = 91 for those aged 85–89 years; NNH = 83 for those aged 90–99 years).

**Table 3:** Fall rates and number needed to harm, by age group and FRD/PIM therapy class count

PIM Class Count	0	1	2	3	4	5	6	7	8	9	10 or More
*Fall Rate (%)*											
Aged 65–74	2.2	2.5	3.3	4.4	5.6	7.6	9.4	12.0	14.6	15.8	19.7
Aged 75–84	5.0	5.5	7.1	8.9	11.0	13.6	15.9	18.2	20.7	19.1	24.8
Aged 85–89	10.5	11.6	13.0	15.3	18.2	21.1	22.6	25.7	25.3	29.8	29.4
Aged 90 or older	16.4	17.6	19.0	20.8	23.7	24.7	26.4	29.0	28.4	26.6	34.8
*NNH* ^*a*^											
Aged 65–74	REF	333	91	45	29	19	14	10	8	7	6
Aged 75–84	REF	200	48	26	17	12	9	8	6	7	5
Aged 85–89	REF	91	40	21	13	9	8	7	7	5	5
Aged 90 or older	REF	83	38	23	14	12	10	8	8	10	5
*N of cases*											
Aged 65–74	249,592	228,484	182,661	119,321	70,219	38,291	20,077	9,663	4,376	1,986	1,228
Aged 75–84	104,926	120,819	115,493	85,382	53,848	29,888	15,200	7,164	3,202	1,247	746
Aged 85–89	23,842	30,491	32,800	25,943	16,479	8,964	4,647	1,972	841	329	194
Aged 90 or older	10,772	13,563	15,362	12,295	8,010	4,301	2,072	869	313	109	46
*90-Day Sensitivity Analysis* ^*b*^											
PIM Class Count	0	1	2	3	4	5	6	7	8 or More		
*Fall Rate (%)*											
Aged 65–74	2.3	3.0	4.0	5.5	7.6	9.9	12.6	15.3	18.4		
Aged 75–84	5.4	6.4	8.2	10.4	13.4	16.3	19.6	21.3	26.1		
Aged 85–89	11.2	12.4	14.3	17.4	20.6	23.1	26.5	28.5	33.2		
Aged 90 or older	17.3	18.4	19.6	22.2	25.9	26.6	33.1	32.9	30.3		
*NNH* ^*a*^											
Aged 65–74	REF	143	59	31	19	13	10	8	6		
Aged 75–84	REF	100	36	20	13	9	7	6	5		
Aged 85–89	REF	83	32	16	11	8	7	6	5		
Aged 90 or older	REF	91	43	20	12	11	6	6	8		
*N of cases*											
Aged 65–74	343,037	254,623	168,391	89,481	41,817	17,748	7122	2467	1212		
Aged 75–84	153,359	146,807	115,483	67,987	32,870	13,899	5046	1742	732		
Aged 85–89	36,324	38,886	33,915	20,754	10,232	4156	1517	513	205		
Aged 90 or older	16,422	17,320	15,835	10,158	5,036	1945	720	210	66		

^a^Multiplicative inverse of absolute difference between fall rate for FRD/PIM therapy class count shown in the row label and fall rate for zero (0) FRD/PIM classes. ^b^Measure FRD/PIM therapy class counts in the 90 days, rather than 365 days, prior to the index date. *FRD* fall risk drug, *NNH* number needed to harm, *PIM* potentially inappropriate medication, *REF* reference category for NNH calculation

Similarly, the risks associated with using 2 to 4 FRD/PIM therapy classes increased in dose-response fashion with age, approximately doubling for those aged 90–99 years compared with those aged 65–74 years (e.g. for 4 FRD/PIM therapy classes, NNH of 29 vs. 14, respectively). Notably, NNH values of < 15, indicating especially high risk (i.e., that treating fewer than 15 adults in that age-FRD/PIM group would result in a fall), were observed with > 6 FRD/PIM classes among those aged 65–74; > 5 FRD/PIM classes among those aged 75–84; and > 4 FRD/PIM classes among those aged 85–99 years. In the sensitivity analysis that measured FRD/PIM counts in the 90 days, rather than 365 days, prior to the index date, rates of falls and the patterns of NNH values were similar, although the number of FRD/PIM classes was smaller overall (Table 3).

In adjusted analyses controlling for medical diagnoses, advancing age was strongly associated with increased fall risk (Table 4). For example, in the equation that assessed the effects of individual medications (Eq. 1), the AOR increased in dose-response fashion across age categories, from 1.753 (95% confidence interval [CI] = 1.723–1.778) for age 75–84 years, to 4.812 (95% CI = 4.579–5.056) for age 95–99 years, relative to the reference category (age 65–74 years). Female sex and previous facility use for a fracture were associated with modest increases in fall risk.

**Table 4:** Logistic regressions of fall events on FRD/PIM measures and demographic characteristics^a^

	Approach to Measurement of Effect of FRD/PIMs								
	Model 1 Individual Medications^b^			Model 2 Counts of FRD/PIM Classes			Model 3 NNH-Based Risk Categorizations		
N of cases included in model	1,678,037			1,678,037			1,678,037		
Model chi-square	108713.4***			105033.2***			88936.9***		
C-statistic (area under ROC curve)	0.774			0.768			0.748		
Nagelkerke R square	0.163			0.158			0.134		
	AOR	CI-L	CI-U	AOR	CI-L	CI-U	AOR	CI-L	CI-U
Age 65–74	REF	REF	REF	REF	REF	REF			
Age 75–84	1.751	1.723	1.778	1.738	1.711	1.765			
Age 85–89	2.830	2.774	2.887	2.802	2.747	2.859			
Age 90–94	3.771	3.674	3.870	3.739	3.644	3.837			
Age 95–99	4.812	4.579	5.056	4.760	4.531	5.000			
Female	1.339	1.321	1.358	1.384	1.365	1.403	1.402	1.383	1.421
Facility use for fracture^c^	1.194	1.159	1.231	1.271	1.233	1.310	1.368	1.328	1.410
Anticholinergic	1.073	1.055	1.092						
Benzodiazepine	1.053	1.035	1.071						
Antidepressant (any type)	1.336	1.314	1.358						
Loop diuretic	1.174	1.154	1.194						
Antimuscarinic	1.160	1.130	1.192						
Antipsychotic, first-generation	1.219	1.115	1.332						
Neuropathic pain reliever	1.092	1.070	1.115						
Opioid pain reliever	1.462	1.439	1.486						
No FRD/PIM classes (NNH reference category)				REF	REF	REF	REF	REF	REF
1 FRD/PIM class				1.021	0.998	1.044			
2 FRD/PIM classes				1.128	1.102	1.154			
3 FRD/PIM classes				1.243	1.213	1.273			
4 FRD/PIM classes				1.357	1.323	1.393			
5 or more FRD/PIM classes				1.579	1.540	1.619			
NNH 200–333							0.850	0.829	0.871
NNH 83–91							1.127	1.098	1.156
NNH 17–48							1.427	1.398	1.456
NNH < 15							1.983	1.934	2.032

^a^All models adjusted for the following diagnoses: Atrial fibrillation, cancer, dementia, depression, diabetes, dizziness/syncope, gait disorder, hepatic impairment, hypertension, neuropathy, orthostatic hypotension, Parkinson’s disease, renal impairment, and substance abuse. Impaired vision was removed in a backwards stepwise logistic regression analysis (P in = 0.001, P out = 0.005) of fall events on diagnosis controlling for age group, sex, and use of a facility for fracture, where diagnoses were measured from 12 months prior through 7 days after the event or proxy date, and facility usage was measured from 12 months prior through 31 days prior to the event or proxy date. ^b^Backwards stepwise analysis of all FRD/PIM classes with unadjusted ORs of 1.50 or more. ^c^Use of a facility for fracture, measured from 12 months prior through 31 days prior to the event or proxy date. *AOR* adjusted odds ratio, *CI-L and CI-U* lower and upper limits, respectively, of the 95% confidence interval, *FRD* fall risk drug, *NNH* number needed to harm, *PIM* potentially inappropriate medication, *REF* reference group, *ROC* receiver operating characteristics

Adjusted results for both equations that measured FRD/PIM use (Table 4, Eqs. 1 and 2) were similar to those produced in bivariate analyses. Specifically, in the backwards stepwise analysis that measured the effects of individual FRD/PIM classes (Model 1), use of opioid pain relievers was associated with the greatest increase in fall risk (AOR = 1.462, 95% CI = 1.439–1.486), followed by antidepressants (AOR = 1.336, 95% CI = 1.314–1.358) and first-generation antipsychotics (AOR = 1.219, 95% CI = 1.115–1.332). In the analysis that measured FRD/PIM use as therapy class counts (Model 2), fall risk increased in dose-response fashion with degree of FRD/PIM use, with the exception that use of a single FRD/PIM class was not significantly associated with fall risk (AOR = 1.021, 95% CI = 0.998–1.044). FRD/PIM-associated increases in the odds of a fall ranged from 13% (AOR = 1.128, 95% CI = 1.102–1.154) with use of 2 classes to 58% (AOR = 1.579, 95% CI = 1.540–1.619) with use of > 5 classes.

The exploratory analysis of age-FRD/PIM interaction (Table 4, Model 3) indicated that the odds of a fall increased in dose-response fashion as NNH category decreased, after adjustment for sex, prior fracture, and fall-related medical diagnoses. Compared with use of no (zero) FRD/PIM classes, age-FRD/PIM class combinations with NNH ranging from 83 to 91 were associated with an increase of 13% (AOR = 1.127, 95% CI = 1.098–1.156) in odds of a fall. For combinations of age and FRD/PIM class count that were identified as high-risk based on NNH < 15, the odds of a fall were approximately doubled (AOR = 1.983, 95% CI = 1.934–2.032). Measures of model fit and quality were similar for the 3 analytic approaches.

## Discussion

This case-control analysis of factors predicting a fall among older adults assessed data from 2013 to 2015, a time period concurrent with the release of the updated 2015 Beers List and 2015 STOPP criteria [8, 9]. A key outcome of the present study was the finding of an interaction between advancing age and FRD/PIM use. Specifically, the degree of fall risk associated with increased use of FRD/PIMs depended on patient age. NNH values < 15, indicating that FRD/PIM treatment of fewer than 15 patients would result in 1 additional fall, compared with no FRD/PIM use, were observed with > 6 FRD/PIM classes among those aged 65–74; > 5 FRD/PIM classes among those aged 75–84; and > 4 FRD/PIM classes among those aged 85–99 years. To the knowledge of these authors, this analysis represents the first large-sample systematic assessment of the degree to which advancing age and degree of FRD/PIM use act jointly in increasing the risk of falls among older adults.

On metrics that have been previously studied, the results of the present study are consistent with those of prior research. Specifically, the risk of a fall increased with older age, even after adjusting for medical diagnoses known to be associated with fall risk [8, 9, 21–29]. Also consistent with previous research was the finding of the present study that fall risk increases with number of FRD/PIM classes utilized by a patient. For example, an increase in fall risk with use of > 2 FRD classes has been noted previously [14, 16]. Similarly, Wallace et al. found an association with increased rates of adverse drug events in patients using > 2 STOPP criteria agents [39]. As in the present study, these studies did not find statistically worse outcomes when only 1 FRD/PIM class was utilized, indicating that the use of multiple FRD/PIM classes may worsen outcomes [16, 39].

In interpreting these findings, it should be noted that the degree of FRD/PIM use observed in the present study was, for a minority of patients, unacceptably high from a clinical perspective. For example, among those aged 90–99 years, those using > 4 FRD/PIM classes represented 23% of patients when measured in the 365 days pre-index and 12% when measured in the 90 days pre-index. Both the marked declines in NNH (i.e., increases in risk) and the known potential for drug interactions associated with increased FRD/PIM use indicate that polypharmacy is a significant concern within this patient population. Because it has previously been observed that use of fall-related medications changes little after a fragility fracture, [40] the present study results may serve as a reminder for the need to conduct medication reviews after a fall event.

Several classes that have previously been implicated in increasing fall risk were also identified as significant predictors of a fall event in the present study sample. These included antipsychotics, benzodiazepines, and antidepressants [8]. One notable finding of the present study was an association of opioid use with an increase in fall risk, estimated at 46% in adjusted analyses. This finding is intriguing, as the STOPP criterion for opioid use in patients with dementia or frequent falls was removed in STOPP version 2. The STOPP criteria do recommend avoiding strong opioids when lower doses or non-opioid pain management therapies would be appropriate treatment options [9]. While the Beers criteria do not include opioids as a FRD/PIM in general recommendations, they recommend avoiding opioid use in patients with history of fall or fractures [8].

These findings should be interpreted in light of U.S. Centers for Disease Control guidance that opioid use may increase risk for fall and fracture in older adults [41]. Notably, a 2015 study by Steinman et al. noted a significant increase in use of opioids in adults aged 65 years or older from 4.1% in 1999–2000 to 9.0% in 2009–2010 [42]. Use of opioids by older adults may place them at increased risk for adverse drug events due to potential drug-drug interactions, renal and/or hepatic dysfunction [43, 44]. As a result, appropriate dose initiation and titration are crucial to reducing these potential side effects in older adults [43, 44].

Our study did not find a significant association between the use of antihypertensives and fall risk. This finding is similar to that of Lipsitz et al., who noted that use of angiotensin converting enzyme inhibitors and calcium channel blockers, even at high doses, was not associated with an increase in fall risk [27]. Bromfield also found that the number of antihypertensive medications was not associated with increased risk of injurious falls [45].

Although the tasks of deprescribing and reducing polypharmacy are the responsibility of all members of the healthcare team, the pharmacist is in a unique position to make recommendations for potential medication discontinuation because of in-depth knowledge of potential drug-drug interactions and dose adjustments for renal or hepatic dysfunction [44]. As evident from the findings of the NNH analysis in the present study, even when it is not possible to discontinue all classes of FRD/PIMs in an older adult due to valid medical indications, [13] a reduction of just 1 or 2 FRD/PIM therapy classes may reduce risk of falls.

A methodological implication of this study is the value of NNH calculations in indicating absolute harm of adverse events in high-risk groups. In the only previous study of joint age-FRD/PIM effects of which we are aware, the investigators used AORs for interaction terms, which indicate relative risk increase [15]. As a measure of treatment-related change in absolute risk, NNH can provide more clinically meaningful information, particularly in modeling costs and benefits of various treatment options, [46] such as when considering referrals for fall prevention services [11, 12, 14].

### Limitations

Several limitations of the present study should be noted. First is the potential for confounding, although the authors attempted to control for various disease states and patient characteristics that may contribute to increased fall risk. Second, the data analyzed were gathered from insurance claims and initially utilized for billing purposes, rather than research purposes. As such, it is possible that “upcoding” or billing/keying errors occurred, although there is no reason to believe that these were more likely to affect any particular age group. Finally, as in any retrospective cohort analysis of claims data, a number of methodological approaches (e.g., codes and time periods) could reasonably have been used to measure outcomes and predictors. Mitigating these concerns are the results of sensitivity analyses and the consistency of the present study findings with those of previous research on this topic.

## Conclusion

Analyses of a Medicare database revealed joint effects of advancing age and degree of FRD/PIM on the risk of falls in older adults. Even small reductions in the number of FRD/PIM classes may reduce the risk of falls, supporting previous calls to “deprescribe” or refer for fall prevention initiatives where appropriate in older patients.

## Additional files

Additional file 1: Medical Claims Codes. (DOCX 15 kb)

Additional file 2: Potentially Inappropriate Medications and Therapy Classes, Study Sample, Observation Period. (DOCX 30 kb)

## Data Availability

The data that support the findings of this study are covered under a license agreement and thus cannot be made public and/or shared. Access to these data is available to any interested party(ies) for a fee set by Truven Health Analytics (https://truvenhealth.com/your-healthcare-focus/government/analytic-research/marketscan/).

## Abbreviations

ACE: Angiotensin converting enzyme
AOR: Adjusted odds ratio
ARB: Angiotensin receptor blocker
CDC: Centers for Disease Control and Prevention
CI: Confidence interval
FRD: Falls-related drug
HIPAA: Health Insurance Portability and Accountability Act
ICD-9: International Classification of Diseases, 9th Revision
IRB: Institutional Review Board
MAOI: Monoamine oxidase inhibitor
NNH: Number needed to harm
OR: Odds ratio
PIM: Potentially inappropriate medication
SNRI: Selective norepinephrine reuptake inhibitor
SSRI: Selective serotonin reuptake inhibitor
STOPP: Screening Tool of Older Persons Potentially Inappropriate Prescriptions
TCA: Tricyclic antidepressant
