# Supplementary material for: Joint effects of advancing age and number of potentially inappropriate medication classes on risk of falls in Medicare enrollees
Source: BMC Geriatr. 2019 Jul 19;19:194. doi: 10.1186/s12877-019-1202-3 (PMC6642496; doi:10.1186/s12877-019-1202-3)
Supplement: Supplementary file 1 — Medical Claims Codes. (DOCX 15 kb) [file 12877_2019_1202_MOESM1_ESM.docx]

**Additional file 1. Medical Claims Codes**

| Diagnosis | Type | Codes and Description |
| --- | --- | --- |
| Atrial fibrillation | ICD-9 | 427.3 Atrial fibrillation and flutter |
| Cancer | ICD-9 | 140.xx-209.xx (malignant neoplasms)  338.3 Neoplasm-related pain  V58.1 Encounter for antineoplastic chemotherapy and immunotherapy |
| Cancer | DRG | 146-148; 374-376; 435-437; 582,583; 597-599; 715,716, 722-724; 736-741; 754-756; 820-825; 834-842; 846, 848 |
| Dementia/mental impairment | ICD-9 | 294.0 Amnestic disorder in conditions classified elsewhere  294.1x Dementia in conditions classified elsewhere  294.2x Dementia, unspecified  331.0x Alzheimer's disease  331.82 Dementia with lewy bodies  290.xx Dementias  291 Alcohol-induced mental disorders  292 Drug-induced mental disorders  294 Persistent mental disorders due to conditions classified elsewhere |
| Depression | ICD-9 | 296.2 Major depressive disorder single episode  296.3 Major depressive disorder recurrent episode  300.4 Dysthymic disorder  311 Depressive disorder, not elsewhere classified |
| Diabetes | ICD-9 | 250 Diabetes mellitus |
| Dizziness | ICD-9 | 780.2 Syncope and collapse  780.4 Dizziness and giddiness |
| Fracture | ICD-9 | 800-804 Fracture of skull  805-809 Fracture of spine and trunk  810-819 Fracture of upper limb  820-829 Fracture of lower limb |
| Gait impairment | ICD-9 | 781.2 Abnormality of gait  781.3 Lack of coordination |
| Hepatic impairment |  | 570 Acute and subacute necrosis of liver  571 Chronic liver disease and cirrhosis  572 Liver abscess and sequelae of chronic liver disease  573 Other disorders of liver |
| Hepatic impairment | DRG | 432-434, 441-443 |
| Hypertension | ICD-9 | 401 Essential hypertension  402 Hypertensive heart disease  403 Hypertensive chronic kidney disease  404 Hypertensive heart and chronic kidney disease  405 Secondary hypertension |
| Hypertension | DRG | 304, 305 |
| Neuropathic pain | ICD-9 | 250.6x Diabetes with neurological manifestations  353 Nerve root and plexus disorders  354 Mononeuritis of upper limb and mononeuritis multiplex  355 Mononeuritis of lower limb and unspecified site  356 Hereditary and idiopathic peripheral neuropathy  357 Inflammatory and toxic neuropathy |
| Orthostatic hypotension | ICD-9 | 458.0 Orthostatic hypotension |
| Parkinsons disease | ICD-9 | 332 Parkinson’s disease |
| Renal impairment | ICD-9 | 585.1 Chronic kidney disease, Stage I)  585.2 Chronic kidney disease, Stage II  585.3 Chronic kidney disease, Stage III  585.4 Chronic kidney disease, Stage IV  585.5 Chronic kidney disease, Stage V  585.6 End stage renal disease  586 Renal failure, unspecified |
| Renal impairment | DRG | 682-685 |
| Renal impairment | Revenue code | 800-809, 822-839, 880-889 |
| Substance abuse | ICD-9 | 291 Alcohol-induced mental disorders  292 Drug-induced mental disorders  303.xx Alcohol dependence syndrome  304.xx Drug dependence  305.xx Nondependent abuse of drugs |
| Substance abuse | DRG | 894-897 |
| Substance abuse | Place of service | 55-57 (substance abuse facility) |
| Vision disorder | ICD-9 | 361 Retinal detachments and defects  365 Glaucoma  366 Cataract  368 Visual disturbances  369 Blindness and low vision  362.5 Degeneration of macula and posterior pole of retina |

DRG=diagnosis-related group; ICD-9=International Classification of Diseases, 9^th^ Revision.
