# Supplementary material for: Joint effects of advancing age and number of potentially inappropriate medication classes on risk of falls in Medicare enrollees
Source: BMC Geriatr. 2019 Jul 19;19:194. doi: 10.1186/s12877-019-1202-3 (PMC6642496; doi:10.1186/s12877-019-1202-3)
Supplement: Supplementary file 2 — Potentially Inappropriate Medications and Therapy Classes, Study Sample, Observation Period. (DOCX 30 kb) [file 12877_2019_1202_MOESM2_ESM.docx]

**Additional file 2. Potentially Inappropriate Medications and Therapy Classes, Study Sample, Observation Period**

| ACE-inhibitors |  | Benazepril | 112861 |
| --- | --- | --- | --- |
|  |  | Captopril | 12382 |
|  |  | Enalapril | 172326 |
|  |  | Fosinopril | 33699 |
|  |  | Lisinopril | 1248006 |
|  |  | Moexipril | 3389 |
|  |  | Perindopril | 3602 |
|  |  | Quinapril | 93802 |
|  |  | Ramipril | 205487 |
|  |  | Trandolapril | 9402 |
|  |  | Total | 1894956 |
| Alpha-blockers |  | Doxazosin | 123661 |
|  |  | Prazosin | 6655 |
|  |  | Terazosin | 98006 |
|  |  | Total | 228322 |
| Alpha2 agonists |  | Clonidine | 146655 |
|  |  | Guanfacine | 6168 |
|  |  | Methyldopa | 3238 |
|  |  | Total | 156061 |
| Antiarrhythmic |  | Disopyramide | 1915 |
| Antidepressant –MAOIs |  | Isocarboxazid | 36 |
|  |  | Phenelzine | 444 |
|  |  | Selegiline | 2846 |
|  |  | Tranylcypromine | 461 |
|  |  | Total | 3787 |
| Antidepressants – other |  | Bupropion | 144443 |
|  |  | Mirtazapine | 121303 |
|  |  | Nefazodone | 1794 |
|  |  | Trazodone | 185518 |
|  |  | Vortioxetine | 1658 |
|  |  | Total | 454716 |
| Antidepressants – SSRIs |  | Citalopram | 270520 |
|  |  | Escitalopram | 230178 |
|  |  | Fluoxetine | 142344 |
|  |  | Fluvoxamine | 3394 |
|  |  | Paroxetine | 134504 |
|  |  | Sertraline | 317655 |
|  |  | Vilazodone | 6420 |
|  |  | Total | 1105015 |
| Antidepressants – Tricyclics |  | Amitriptyline | 105361 |
|  |  | Amoxapine | 435 |
|  |  | Clomipramine | 1682 |
|  |  | Desipramine | 3020 |
|  |  | Doxepin | 23501 |
|  |  | Imipramine | 12871 |
|  |  | Nortriptyline | 34764 |
|  |  | Protriptyline | 436 |
|  |  | Trimipramine | 127 |
|  |  | Total | 182197 |
| Antidepressants –SNRIs |  | Desvenlafaxine | 16272 |
|  |  | Duloxetine | 181968 |
|  |  | Levomilnacipran | 909 |
|  |  | Milnacipran | 3950 |
|  |  | Venlafaxine | 139885 |
|  |  | Total | 342984 |
| Antiemetic/antipsychotic |  | Prochlorperazine | 18506 |
| Antiemetic |  | Promethazine | 35138 |
| Antihistamines |  | Brompheniramine | 3381 |
|  |  | Carbinoxamine | 670 |
|  |  | Chlorpheniramine | 22714 |
|  |  | Clemastine | 694 |
|  |  | Cyproheptadine | 5270 |
|  |  | Dexbrompheniramine | 13 |
|  |  | Dexchlorpheniramine | 5 |
|  |  | Diphenhydramine | 1452 |
|  |  | Doxylamine | 4 |
|  |  | Hydroxyzine | 52708 |
|  |  | Meclizine | 76317 |
|  |  | Total | 163228 |
| Antimuscarinics |  | Darifenacin | 19161 |
|  |  | Fesoterodine | 23657 |
|  |  | Flavoxate | 634 |
|  |  | Oxybutynin | 148955 |
|  |  | Solifenacin | 87807 |
|  |  | Tolterodine | 69766 |
|  |  | Trospium | 14464 |
|  |  | Total | 364444 |
| Antiparkinson |  | Benztropine | 6091 |
|  |  | Trihexyphenidyl | 3123 |
|  |  | Total | 9214 |
| Antispasmodics |  | Atropine | 41922 |
|  |  | Dicyclomine | 44109 |
|  |  | Homatropine | 22149 |
|  |  | Hyoscyamine | 24480 |
|  |  | Propantheline | 85 |
|  |  | Scopolamine | 7679 |
|  |  | Total | 140424 |
| ARB |  | Candesartan | 33456 |
|  |  | Eprosartan | 954 |
|  |  | Irbesartan | 84727 |
|  |  | Losartan | 711613 |
|  |  | Telmisartan | 34722 |
|  |  | Valsartan | 228300 |
|  |  | Total | 1093772 |
| Benzodiazepines |  | Alprazolam | 417307 |
|  |  | Chlordiazepoxide | 17683 |
|  |  | Clobazam | 124 |
|  |  | Clonazepam | 169061 |
|  |  | Clorazepate | 12538 |
|  |  | Diazepam | 106471 |
|  |  | Estazolam | 1998 |
|  |  | Flurazepam | 3175 |
|  |  | Lorazepam | 256858 |
|  |  | Midazolam | 35 |
|  |  | Oxazepam | 5301 |
|  |  | Quazepam | 5 |
|  |  | Temazepam | 93147 |
|  |  | Triazolam | 12826 |
|  |  | Total | 1096529 |
| Beta-blockers |  | Acebutolol | 7720 |
|  |  | Atenolol | 504303 |
|  |  | Betaxolol | 6027 |
|  |  | Bisoprolol | 85546 |
|  |  | Carteolol | 1620 |
|  |  | Nadolol | 27395 |
|  |  | Penbutolol | 186 |
|  |  | Pindolol | 4031 |
|  |  | Propranolol | 92783 |
|  |  | Timolol | 113759 |
|  |  | Total | 843370 |
| Calcium channel blockers |  | Amlodipine | 1568582 |
|  |  | Diltiazem | 356299 |
|  |  | Felodipine | 36895 |
|  |  | Isradipine | 2530 |
|  |  | Nicardipine | 838 |
|  |  | Nifedipine | 133728 |
|  |  | Nisoldipine | 10527 |
|  |  | Verapamil | 125510 |
|  |  | Total | 2234909 |
| Central acting |  | Reserpine | 9 |
| First-generation antipsychotic |  | Chlorpromazine | 1893 |
|  |  | Droperidol | 2 |
|  |  | Fluphenazine | 1123 |
|  |  | Haloperidol | 8064 |
|  |  | Loxapine | 441 |
|  |  | Perphenazine | 1525 |
|  |  | Pimozide | 191 |
|  |  | Thioridazine | 1031 |
|  |  | Trifluoperazine | 1212 |
|  |  | Total | 15482 |
| Hypnotics |  | Eszopiclone | 30689 |
|  |  | Zaleplon | 9297 |
|  |  | Zolpidem | 394348 |
|  |  | Total | 434334 |
| Loop diuretics |  | Bumetanide | 50133 |
|  |  | Furosemide | 838762 |
|  |  | Torsemide | 56696 |
|  |  | Total | 945591 |
| Opioid analgesic |  | Buprenorphine | 7437 |
|  |  | Butalbital | 1 |
|  |  | Butorphanol | 842 |
|  |  | Codeine | 82453 |
|  |  | Dihydrocodeine | 2 |
|  |  | Fentanyl | 71527 |
|  |  | Hydrocodone | 7829 |
|  |  | Hydromorphone | 21930 |
|  |  | Levorphanol | 89 |
|  |  | Meperidine | 2151 |
|  |  | Methadone | 12732 |
|  |  | Morphine | 47186 |
|  |  | Nalbuphine | 105 |
|  |  | Oxycodone | 134902 |
|  |  | Oxymorphone | 6081 |
|  |  | Paregoric | 122 |
|  |  | Tapentadol | 7584 |
|  |  | Tramadol | 404496 |
|  |  | Total | 807469 |
| Other – neuropathic pain |  | Gabapentin | 502740 |
|  |  | Pregabalin | 119195 |
|  |  | Total | 621935 |
| Potassium sparing diuretics |  | Amiloride | 9669 |
|  |  | Eplerenone | 12025 |
|  |  | Spironolactone | 181903 |
|  |  | Triamterene | 1387 |
|  |  | Total | 204984 |
| Second-generation antipsychotics |  | Aripiprazole | 27175 |
|  |  | Asenapine | 602 |
|  |  | Clozapine | 2478 |
|  |  | Iloperidone | 204 |
|  |  | Lurasidone | 1710 |
|  |  | Olanzapine | 27756 |
|  |  | Paliperidone | 767 |
|  |  | Quetiapine | 96725 |
|  |  | Risperidone | 42287 |
|  |  | Ziprasidone | 4096 |
|  |  | Total | 203800 |
| Skeletal muscle relaxants |  | Cyclobenzaprine | 126730 |
|  |  | Orphenadrine | 4900 |
|  |  | Total | 131630 |
| Thiazide like diuretics |  | Chlorothiazide | 1025 |
|  |  | Chlorthalidone | 58400 |
|  |  | Hydrochlorothiazide | 684865 |
|  |  | Indapamide | 29005 |
|  |  | Metolazone | 33178 |
|  |  | Total | 806473 |
| Vasodilators |  | Hydralazine | 132284 |
|  |  | Minoxidil | 7879 |
|  |  | Total | 140163 |

ACE=angiotensin-converting enzyme; ARB=angiotensin receptor blocker; MAOI=monoamine oxidase inhibitor; SNRI=selective norepinephrine reuptake inhibitor; SSRI=selective serotonin reuptake inhibitor.
